# The neuroprotective properties of palmitoylethanolamine against oxidative stress in a neuronal cell line

**DOI:** 10.1186/1750-1326-4-50

**Published:** 2009-12-10

**Authors:** R Scott Duncan, Kent D Chapman, Peter Koulen

**Affiliations:** 1 Departments of Basic Medical Science and Ophthalmology, University of Missouri - Kansas City, 2411 Holmes St, Kansas City, MO 64108-2792, USA; 2 Department of Biological Sciences, University of North Texas, 1155 Union Circle, #305220, Denton, TX 76203-5217, USA; 3 Center for Plant Lipid Research, University of North Texas, 1155 Union Circle, #305220, Denton, TX 76203-5217, USA

## Abstract

**Background:**

N-acylethanolamines (NAEs) are lipids upregulated in response to cell and tissue injury and are involved in cytoprotection. Arachidonylethanolamide (AEA) is a well characterized NAE that is an endogenous ligand at cannabinoid and vanilloid receptors, but it exists in small quantities relative to other NAE types. The abundance of other NAE species, such as palmitoylethanolamine (PEA), together with their largely unknown function and receptors, has prompted us to examine the neuroprotective properties and mechanism of action of PEA. We hypothesized that PEA protects HT22 cells from oxidative stress and activates neuroprotective kinase signaling pathways.

**Results:**

Indeed PEA protected HT22 cells from oxidative stress in part by mediating an increase in phosphorylated Akt (pAkt) and ERK1/2 immunoreactivity as well as pAkt nuclear translocation. These changes take place within a time frame consistent with neuroprotection. Furthermore, we determined that changes in pAkt immunoreactivity elicited by PEA were not mediated by activation of cannabinoid receptor type 2 (CB2), thus indicating a novel mechanism of action. These results establish a role for PEA as a neuroprotectant against oxidative stress, which occurs in a variety of neurodegenerative diseases.

**Conclusions:**

The results from this study reveal that PEA protects HT22 cells from oxidative stress and alters the localization and expression levels of kinases known to be involved in neuroprotection by a novel mechanism. Overall, these results identify PEA as a neuroprotectant with potential as a possible therapeutic agent in neurodegenerative diseases involving oxidative stress.

## Introduction

*N*-Acylethanolamines (NAEs) are endogenous lipids involved in cell signaling and they are synthesized in response to cellular injury [1,2]. The NAE, arachidonylethanolamide (AEA), is a cannabinoid exhibiting cytoprotective properties against a wide variety of pathological insults including excitotoxicity, oxidative stress and hypoxia [3-10]. Cannabinoids activate the G-protein-coupled cannabinoid receptors (CB1 and CB2) leading to downregulation of PKA and activation of the ERK MAPK pathway, a neuroprotective signaling pathway [11-18]. Furthermore, the activation of Akt by cannabinoids further supports their role as neuroprotectants [16]. Interestingly, concentrations of AEA in various tissues including the brain are relatively low compared to other NAE species such as the non-cannabinoid NAE, palmitoylethanolamine (PEA) [19,20].

Some saturated and monounsaturated NAEs have been shown to activate ERK1/2 phosphorylation pathway through a CB1-independent mechanism [21]. Interestingly, the yeast *Saccharomyces cerevisiae*, which does not express cannabinoid or vanilloid receptors, synthesizes various NAE species in response to oxidative stress [22]. This result further substantiates a non-cannabinoid receptor- and a non-vanilloid receptor-mediated function for some NAEs.

In the present study, we determined that the lipid PEA is neuroprotective against oxidative insult. PEA treatment can activate the ERK1/2 MAP kinase and Akt proteins as determined by microfluorimetric measurements. Here, we identified that PEA can increase ERK1/2 and Akt phosphorylation and nuclear translocation of phospho-Akt (Ser473) (pAkt) which suggests that the neuroprotective effects of PEA may be mediated, in part, by activation of these kinases. Furthermore, we provide evidence that this effect of PEA is not mediated through the activation of CB2. The results of the present study identify PEA as a potential therapeutic agent for the treatment of neurodegenerative diseases in which oxidative stress occurs. Furthermore, PEA shares a similar mechanism of action with other neuroprotectants providing further evidence for the importance of kinase signaling in neuroprotection.

## Materials and methods

### Chemicals

Palmitoylethanolamine (PEA), JWH-015, AM-1242 and AM-630 were purchased from Alexis Biochemicals (Switzerland). Calcein-acetoxymethyl ester (calcein-AM) was purchased from Alexis Biochemicals or EMD/Calbiochem. Tert-butylhydroperoxide (tBHP) was purchased from Acros Organics (Belgium).

### Cell culture

The murine hippocampal cell line HT22 was cultured as described previously [23]. In brief, HT22 cells were grown in Dulbecco's modified Eagle's medium (DMEM) with high glucose and 1 mM sodium pyruvate (Mediatech), 2 mM Glutamax (Invitrogen), 5% bovine growth serum (BGS) (Hyclone) and penicillin-streptomycin (Mediatech). Cultures were kept at a confluency of less than 70% during the culturing process. For immunofluorescence analysis, HT22 cells were plated on poly-L-lysine-coated 12 mm coverslips overnight followed by treatments as described in the text. Immunocytochemistry was subsequently conducted as described elsewhere in detail [23].

### Assessment of cell viability

Oxidative stress was induced by exposing cells to 20 - 25 μM tBHP. The fluorimetric calcein- AM and VYBRANT glucose-6-phosphate dehydrogenase (G-6-PD) cytotoxicity assays (Invitrogen) were conducted in 96 well plates in order to assess cell viability in a high-throughput format. All 96 well plate assays for HT22 cell viability were conducted using a cell density of 2,000 cells/well unless noted otherwise. For the calcein-AM assay, media was removed from plates after 16 - 20 hours of tBHP exposure followed by replacement with Hank's balanced salt solution (HBSS) with 2 mM CaCl_2 _and calcein-AM dye at a final concentration of 4 μM for 20 minutes to load cells. Calcein fluorescence was measured using a fluorimetric plate reader (Perkin-Elmer Victor^3^) with the appropriate filters (485 nm excitation and 530 nm emission). The underlying mechanism is that viable cells take up the ester form of calcein (calcein-AM) and convert it to the non-ester form, calcein. Calcein accumulates in viable cells resulting in elevated fluorescence. The VYBRANT G-6-PD cytotoxicity assays were conducted 10 - 12 hours after tBHP exposure according to the manufacturer's instructions with a substrate reaction time of 5 - 6 hours at 37°C and read at 530 nm excitation and 560 nm emission. In principle, non-viable cells leak their contents into the culture media thus allowing for the assay of enzyme activity, such as G-6-PD activity. All raw data was analyzed, normalized and graphed in Microscoft Excel.

### Immunocytochemistry after PEA treatment

HT22 cells were plated on poly-L-lysine-coated 12 mm coverslips at 40,000 cells/ml and maintained for 24 hours. The media was removed and replaced with media containing 100 μM PEA (or ethanol vehicle) for various time points (as described in the results). After the PEA exposure, the cells were rinsed and fixed with 4% paraformaldehyde followed by immunocytochemistry (ICC) using polyclonal sera raised against Akt, pAkt, ERK1/2, phospho-ERK1/2 (Thr202/Tyr204) (pERK1/2), p38 or monoclonal rabbit anti-phospho-p38 (Thr180/Tyr182) antibody (Cell Signaling Technology, Danvers, MA) using a method described elsewhere [23]. After completion of ICC and mounting, images were acquired at 20× magnification using an Olympus IX70 fluorescence microscope. TIFF images were analyzed in Simple PCI by selecting three (3) background regions of interest (ROIs) followed by nuclear then cytosolic ROIs for each cell. The nuclear and cytosolic data was separated in Microsoft Office Excel and graphed.

### Statistics

For neuroprotection experiments (calcein-AM and VYBRANT cytotoxicity assays), a one-way ANOVA with a Neumann-Keuls post-hoc test was conducted using GraphPad Prism 5.01. For immunofluorescence experiments, an F-test was conducted in Microsoft Excel between an individual treatment group and its respective untreated control group to determine which type of T-test should be used for group comparisons. The mean fluorescence intensity from each treatment group was separately compared to the mean fluorescence intensity of the untreated control group using a two-sample T-test with either equal or unequal variances. Multiple comparisons were not done with the T-test. A P-value of less than or equal to 0.05 was considered significant.

## Results

### PEA protects HT22 from oxidative stress

**Figure 1:**
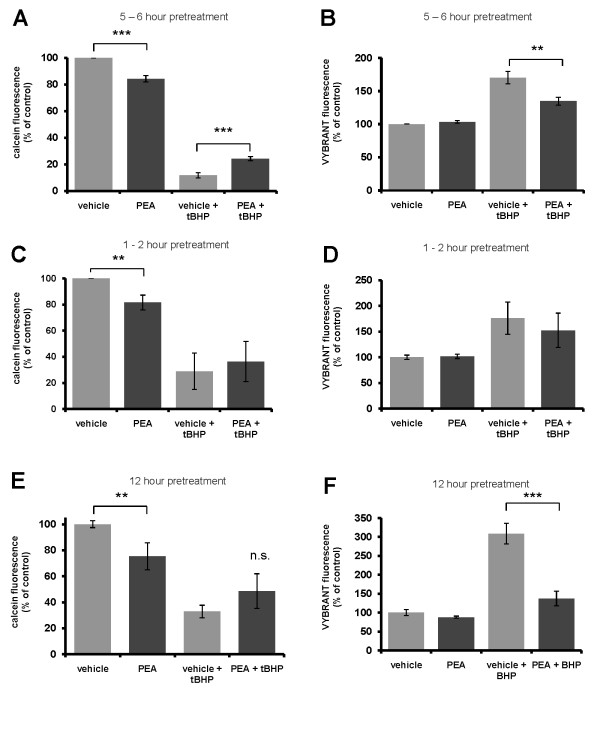
**PEA pretreatment protects HT22 cells from tBHP exposure**. (A, B), HT22 cells were pretreated with 100 μM PEA for 5 - 6 hours prior to an overnight (10 - 12 hour or 16 - 20 hour) tBHP exposure. PEA pretreatment led to a significant neuroprotection against oxidative stress as determined by an increase in calcein fluorescence (A), and a reduction in fluorescent product from G-6-PD in the cell culture media (B). For this study, *n *equals three (3) experiments. HT22 cells were pretreated with PEA for 1 - 2 hours (C, D) or 12 hours (E, F) prior to tBHP exposure. PEA pre-treatment for 1 - 2 hours had no effect on viability (E), whereas pretreatment for 12 hours reduced measured extracellular G-6-PD activity (F). A P-value of ≤ 0.05, ≤ 0.01 and ≤ 0.001 is indicated by *, ** and ***, respectively, as determined by a Newman-Keuls multiple comparison test.

HT22 cells were treated with PEA (100 μM) for various time periods to determine the therapeutic window for PEA. Use of PEA concentrations lower than 100 μM do not offer protection of HT22 cells from tBHP-mediated oxidative stress and, therefore, these data are not included. PEA treatment for 5 - 6 hours prior to overnight (16 - 20 hour) tBHP exposure significantly protects HT22 cells from tBHP as indicated by an increase in calcein fluorescence and a decrease in G-6-PD activity (VYBRANT reagent fluorescence) (Fig. 1A, B). Treatment of cells with PEA for shorter time periods (1 - 2 h) prior to tBHP insult offered no neuroprotection (Fig. 1C, D) while a longer time period (12 h) prior to tBHP exposure exhibit a significant reduction in markers of cell death according to preliminary data (Fig. 1E, F). This suggests that the therapeutic window of PEA treatment before insult is critical for its neuroprotective properties.

### PEA treatment increases pAkt kinase immunoreactivity and controls nuclear translocation by a CB2-independent mechanism

**Figure 2:**
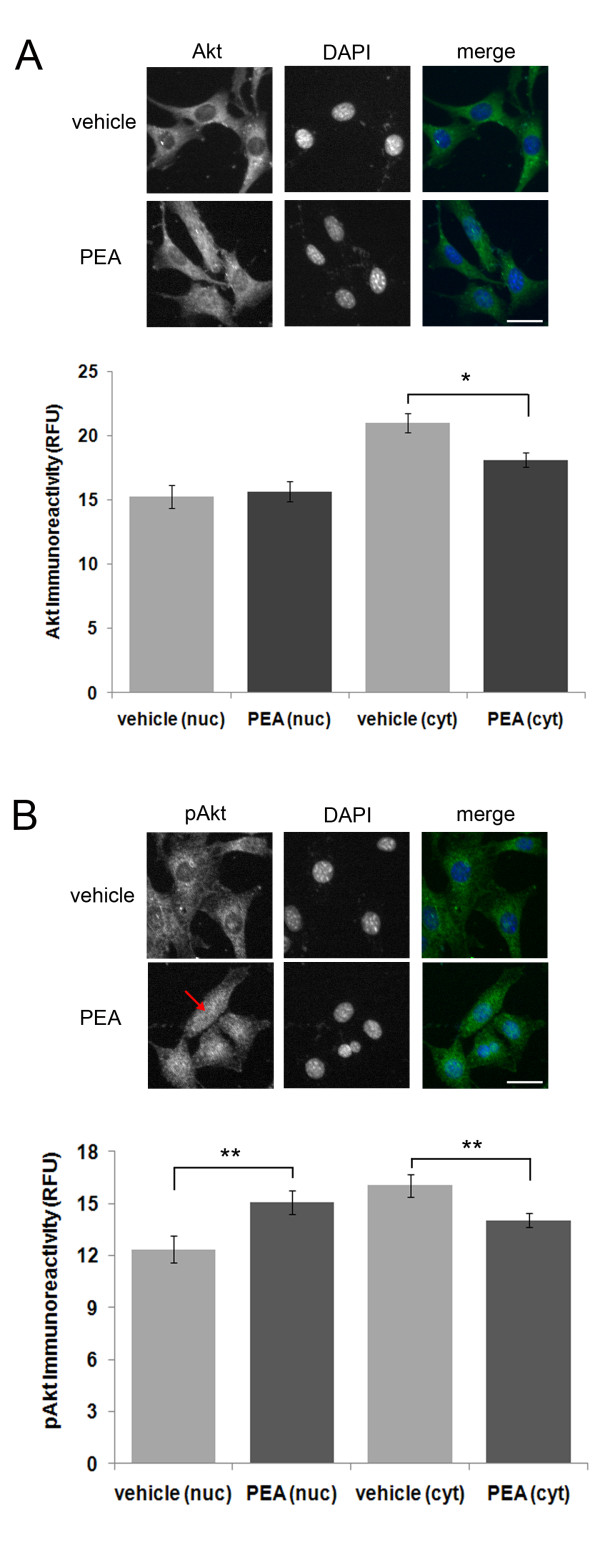
**PEA treatment of HT22 cells leads to an increase in nuclear pAkt immunoreactivity**. (A), a four (4) hour PEA treatment led to a significant decrease in Akt immunoreactivity but it had no effect on nuclear Akt immunoreactivity. For the treatment groups, *n *equals 50 and 66 cells for vehicle and PEA treatments, respectively. (B), a four (4) hour PEA treatment led to a significant increase in nuclear pAkt (red arrow) and a significant decrease in cytosolic pAkt. For the treatment groups, n equals 56 and 75 cells, respectively. A P-value of <0.05 and ≤ 0.01 is indicated by * and **, respectively, as determined by a two-sample t-test. The white scale bar is 25 μm.

Exposure of HT22 cells to PEA for four hours had no significant effect on nuclear Akt immunoreactivity (Fig. 2A), but it resulted in a significant increase in nuclear pAkt immunoreactivity (Fig. 2B). A six hour PEA treatment also had the same effect (data not shown).

**Figure 3:**
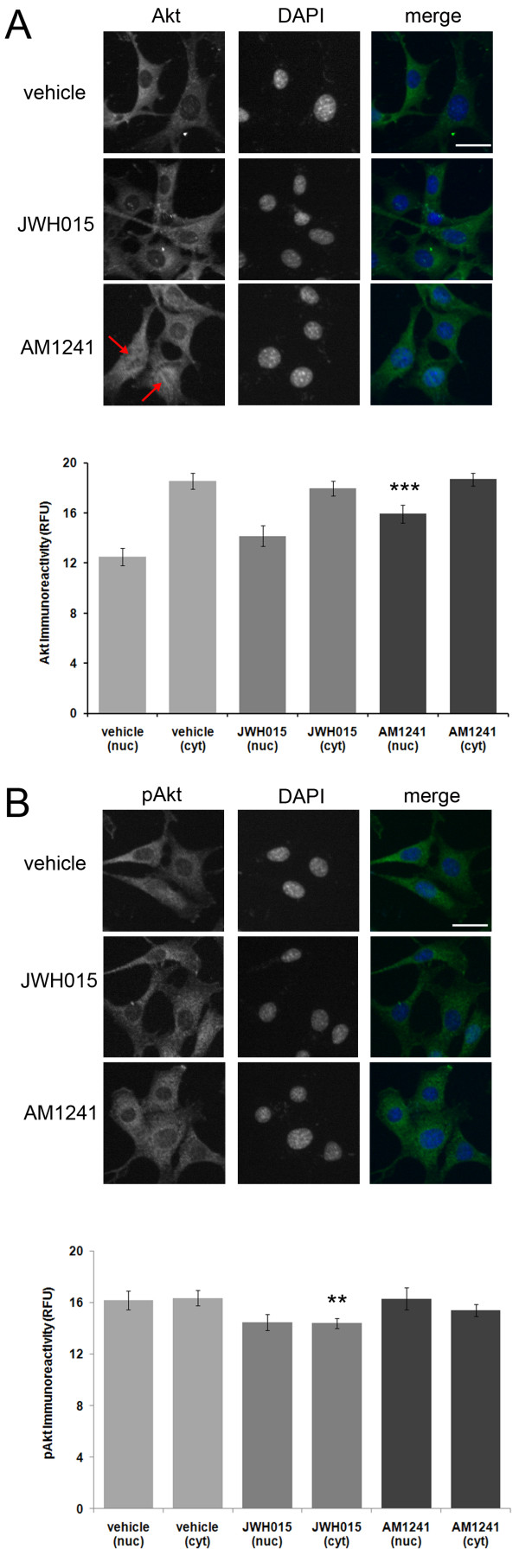
**The CB2 agonist AM1241, but not JWH-015, increases nuclear Akt immunoreactivity**. (A), Treatment of HT22 cells with the CB2 agonist JWH-015 had no effect on nuclear Akt immunoreactivity. Treatment with AM1241, however, led to an increase in nuclear Akt immunoreactivity (red arrows). (B), Treatment of HT22 cells with the CB2 agonists JWH-015 and AM1241 had no effect on nuclear or cytosolic pAkt immunoreactivity. In fact, JWH-015 led to a reduction in pAkt immunoreactivity. For the Akt study, *n *equals 61, 56 and 59 cells for vehicle, JWH-015 and AM-1241 treatments, respectively. For the pAkt study, *n *equals 80, 91 and 64 cells for vehicle, JWH-015 and AM-1241 treatments, respectively. A P-value of < 0.01 and ≤ 0.001 is indicated by ** and ***, respectively, as determined by a two-sample t-test. The white scale bar is 25 μm.

To determine whether or not PEA's effects on Akt phosphorylation and nuclear translocation required activation of CB2, HT22 cells were treated with the CB2 agonists, JWH-015 and AM1241, for 6 hours prior to Akt and pAkt immunolabeling. Treatment of HT22 cells with 10 μM JWH-015 alone had no effect on nuclear or cytosolic Akt immunoreactivity (Fig. 3A) but it led to a decrease in cytosolic pAkt immunoreactivity (Fig. 3B). Interestingly, activated Akt (pAkt) has cytosolic functions distinct from its nuclear functions [24,25]. Treatment of cells with 10 μM AM1241 alone led to a significant increase in nuclear Akt immunoreactivity (Fig. 3A), but it had no effect on pAkt immunoreactivity (Fig. 3B). Our data suggest that JWH-105 fails to mimic the effects of PEA on pAkt immunoreactivity in HT22 cells. This suggests that PEA's ability to increase nuclear pAkt is through a CB2-independent mechanism.

**Figure 4:**
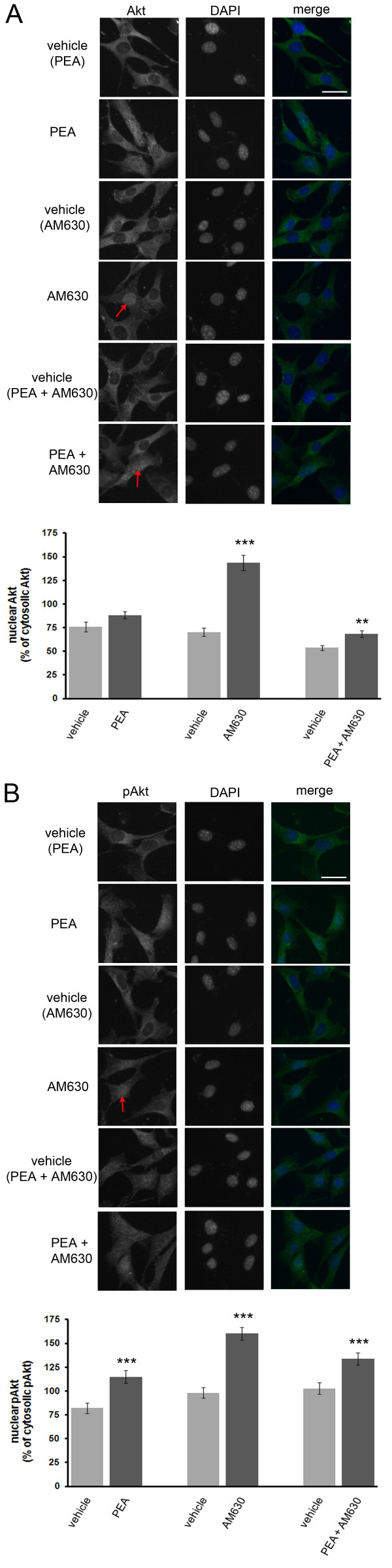
**The CB2 antagonist, AM630, alters Akt and pAkt immunoreactivity in HT22 cells**. (A), Treatment of HT22 cells with AM630 leads to an increase in nuclear Akt immunoreactivity (red arrow) and an increase in the nuclear over cytosolic Akt immunoreactivity ratio (graph) while the presence of PEA reduces this increase (graph). (B), Likewise AM630 increases nuclear pAkt immunoreactivity (red arrow) and increases the nuclear over cytosolic pAkt immunoreactivity ratio (graph) and while the inclusion of PEA reduces this effect (graph). Co-treatment of cells with both PEA and AM630 results in an increase in nuclear Akt (red arrow, graph). For the Akt study, *n *equals 50, 66, 52, 53, 62 and 65 cells for vehicle (PEA), PEA, vehicle (AM630), AM630, vehicle (PEA and AM630) and PEA and AM630 treatments, respectively. For the pAkt study, *n *equals 56, 75, 94, 78, 75 and 89 cells for vehicle (PEA), PEA, vehicle (AM630), AM630, vehicle (PEA and AM630) and PEA and AM-630 treatments, respectively. A P-value of ≤ 0.05, < 0.01 and ≤ 0.001 is indicated by *, ** and ***, respectively, as determined by a two-sample t-test.

In addition, the CB2 antagonist, AM630 was utilized to rule out CB2 activation in PEAs effects on Akt and pAkt (Fig. 4). Although a 6 hour treatment with PEA had no significant effect on Akt immunoreactivity, treatment with AM630 led to a significant increase in nuclear Akt relative to cytosolic Akt (Fig. 4A). Interestingly, combined treatment with PEA and AM630 only led to a slight increase in nuclear Akt immunoreactivity relative to cytosolic Akt (Fig. 4A).

A 6 hour treatment of cells with AM630 led to a significant increase in nuclear pAkt immunoreactivity relative to cytosolic pAkt immunoreactivity similar to that observed for PEA-treated cells, indicating that PEAs effects were not mediated through CB2 receptor activation (Fig. 4B). Interestingly, combined treatment with PEA and AM630 led to an increase in nuclear pAkt relative to cytosolic pAkt immunoreactivity in part due to a decrease in cytosolic pAkt immunoreactivity. These results suggest that alterations in Akt and pAkt compartmentalization are affected differently by PEA and AM630. These results provide evidence that CB2 activation is not responsible for the observed changes in pAkt immunoreactivity mediated by PEA treatment in HT22 cells.

### Effect of PEA treatment on MAPK and phosphorylated MAPK immunoreactivity

**Figure 5:**
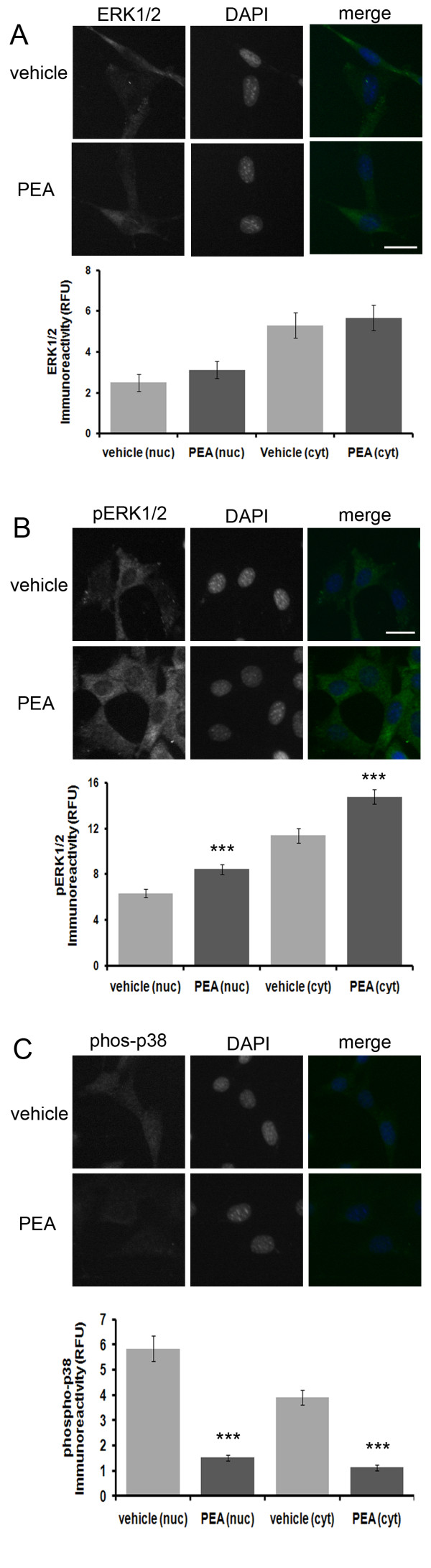
**PEA increases nuclear and cytosolic pERK1/2 and decreases nuclear and cytosolic phospho-p38 immunoreactivity in HT22 cells independent of CB2 activation**. (A), A brief, 30 minute PEA treatment of HT22 cells has no effect on nuclear or cytosolic ERK1/2 immunoreactivity. (B), a 30 minute PEA treatment results in a transient increase in nuclear and cytosolic pERK/12 immunoreactivity. In the ERK1/2 immunofluorescence experiment, *n *equals 24 and 34 cells for vehicle and PEA treatments, respectively. In the pERK1/2 immunofluorescence experiment, *n *equals 46 and 81 cells for vehicle and PEA treatments, respectively. A P-value of ≤ 0.001 is indicated by *** as determined by a two-sample t-test. (C), Treatment of HT22 cells with PEA for 30 min. resulted in a dramatic and significant decrease in both nuclear and cytosolic phospho-p38 immunoreactivity. In this study, for the 30 minute treatment group, *n *equals 42 and 35 cells for vehicle and PEA treatments, respectively. For the 60 minute treatment group, *n *equals 50 and 41 cells for vehicle and PEA treatments, respectively. A P-value of ≤ 0.001 is indicated by *** as determined by a two-sample t-test.

**Figure 6:**
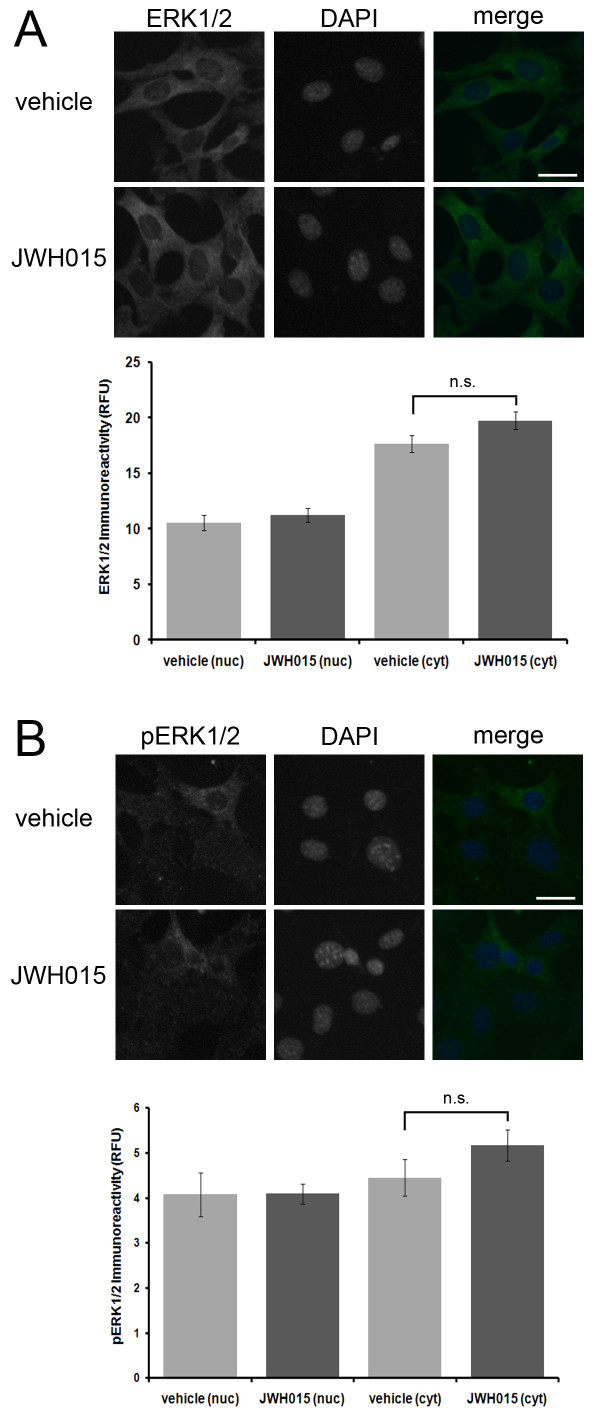
**The CB2 agonist JWH-015 has no effect on ERK1/2 or pERK1/2 immunoreactivity in HT22 cells**. A brief 60 minute treatment of HT22 cells with the CB2 agonist JWH-015 fails to significantly increase ERK1/2 immunoreactivity (A) or pERK1/2 immunoreactivity (B). In the ERK1/2 immunofluorescence experiment, *n *equals 54 and 59 cells for vehicle and JWH-015 treatments, respectively. In the pERK1/2 immunofluorescence experiment, *n *equals 34 and 40 cells for vehicle and JWH-015 treatments, respectively. Significance was determined by a two-sample t-test.

Exposure of HT22 cells to PEA for 30 minutes had no effect on ERK1/2 immunoreactivity (Fig. 5A). Exposure of cells to PEA for 30 minutes, however, led to a significant increase in nuclear and cytosolic pERK1/2 immunoreactivity (Fig. 5B). Exposure of cells to PEA for 60 minutes resulted in a dramatic and significant decrease in both nuclear and cytosolic phospho-p38 immunoreactivity (Fig. 5C). Furthermore, treatment of HT22 cells with JWH015 had no significant effect on ERK1/2 or pERK1/2 immunoreactivity (Fig. 6A, B). This suggests that PEAs effects on ERK1/2 and pERK1/2 immunoreactivity are not due to CB2 activation.

## Discussion

From these studies, we conclude that PEA (at 100 μM) protects HT22 cells from oxidative stress when cells are pretreated for 5 - 6 hours prior to tBHP exposure (Fig. 1). Interestingly, shorter PEA pretreatment times did not protect and PEA pretreatment for 12 hours protected cells from tBHP insult as measured by G-6-PD activity in the culture media (Fig. 1). These studies identify PEA as a neuroprotectant that is naturally synthesized in neurons.

In addition, we provide evidence that PEA treatment facilitates the nuclear translocation of pAkt in a neuronal cell line by a CB2-independent mechanism (Fig. 3 and 4). Furthermore, we determined that PEA leads to a rapid and transient increase in nuclear and cytosolic pERK1/2, but not ERK1/2 (Fig. 5). This mechanism is independent of CB2 activation as it could not be mimicked by the CB2 agonist, JWH-015 (Fig. 6). In addition, we determined that PEA exposure leads to a significant reduction in nuclear and cytosolic phospsho-p38 immunoreactivity in HT22 cells (Fig. 5C). These effects are within the timeframe required to cause neuroprotection in HT22 cells. Taken together, these data suggest that PEA activates kinases known to be involved in neuroprotective signaling, thus providing a possible mechanism by which NAEs protect neurons.

Cannabinoids, such as AEA, exhibit neuroprotective properties against a wide variety of pathological insults including excitotoxicity, oxidative stress and hypoxia through the activation of CB1 [2-10]. Cannabinoids activating CB1 and CB2 can subsequently activate the ERK1/2, p38 and JNK MAPKs in addition to Akt [14-16,26-30].

MAPKs and Akt initiate neuroprotective responses [31-35]. For example, in HT22 cells, short-term activation of ERK1/2 is involved in a cellular adaptive response to glutamate toxicity [34]. In PC12 cells, H2O2 treatment leads to the rapid phosphorylation of ERK1/2 and p38 [36]. Cannabinoid activation of CB1 and CB2 receptors leads to downregulation of PKA and activation of the ERK MAPK pathway, a neuroprotective signaling pathway [14,15,26,33-35]. The data presented here provide evidence that PEA, which is neuroprotective, can elevate pERK1/2 and reduce phospho-p38 immunoreactivity in HT22 cells providing evidence for a possible mechanism of action for PEA mediated neuroprotection.

The activation of Akt further supports a role for cannabinoids as neuroprotectants [16,30]. In neurons, Akt activation results in neuroprotection by inhibiting pro-apoptotic proteins including Bad, FOXO, GSK3α/β and caspase-9 [32]. Akt activation can inhibit FOXO- and p53-mediated transcription of death genes such as FasL and Bax [17]. Activated Akt (pAkt) has also been shown to activate NFκB- and CREB-mediated transcription leading to protection of culture cells against serum deprivation [37,38]. It is not clear, however, whether inhibition of pro-apoptotic or activation of anti-apoptotic transcription factors occurs after pAkt is translocated to the nucleus. The nuclear translocation of Akt in response to PEA treatment occurring within a time frame consistent with neuroprotection PEA suggests a possible mechanism involving transcription of neuroprotective genes. We previously showed that inositol 1, 4, 5-trisphosphate (IP3) receptors located in the cytosolic compartment (endoplasmic reticulum) can are phosphorylated by activated Akt thus leading to an increase in activity [24,25]. It is possible, therefore, that PEA activation of Akt in the cytosolic compartment may lead to IP3 receptor phosphorylation and activity. This activity Akt may have a role in neuroprotective signaling in addition to the nuclear functions of pAkt.

Studies in immune cells reveal that PEA has CB2 receptor-independent effects [39]. Several NAEs including PEA lead to increase ERK phosphorylation and AP-1 activity in mouse JB6 epidermal cells [21]. The CB1 agonist Win 55212, however, could not stimulate ERK phosphorylation or AP-1 activation suggesting a CB1-independent function of NAEs in cell signaling and gene transcription [21].

Since saturated NAEs, such as PEA, do not bind CB1 and exhibit poor affinity for CB2, we hypothesized that these NAEs exhibit neuroprotective properties by a mechanism independent of CB2 [40,41]. To rule out CB2-mediated effects in PEA neuroprotective signaling, we measured the effect of CB2 agonists on Akt/pAkt and ERK/pERK immunoreactivity. The CB2 agonist, JWH-015 had no effect on nuclear Akt or pAkt immunoreactivity in HT22 cells. The CB2 agonist AM1241, however, increased nuclear Akt immunoreactivity, but it had no effect on pAkt immunoreactivity. Together, these data suggest that PEAs effect on pAkt were not mediated through CB2 activation. Further evidence for this comes from the observation that treatment of cells with the CB2 antagonist, AM630, mimics instead of inhibits the effects of PEA on cytosolic Akt immunoreactivity and nuclear and cytosolic pAkt immunoreactivity in HT22 cells. These observations using AM630 suggest that either AM630 inverse agonist activity at CB2 receptors may lead to an increase in nuclear pAkt immunoreactivity or that AM630 may have a yet unknown receptor that alters pAkt activity upon activation. Given the reported weak partial agonist activity of PEA at CB2 receptors [40,41] and the inverse agonist activity of AM630 at CB2 receptors [42], it is unlikely that the similar effects between PEA and AM630 on pAkt are due to a CB2-dependent mechanism.

The present study identifies PEA as a neuroprotectant exerting its actions through a mechanism not involving classical cannabinoid receptors and through signaling pathways known to be involved in a neuroprotective response. The present studies lay the groundwork for better understanding the potential neuroprotective effects that non-cannabinoid NAEs have in neurodegenerative diseases.

## Competing interests

KDC and PK have patents pending relating to the contents of the manuscript. RSD declares no competing interests.

## Authors' contributions

RSD designed experiments, conducted experiments, acquired and analyzed data, drafted and co-authored the manuscript. KDC and PK designed experiments, analyzed data and co-authored the manuscript. All authors reviewed the final version of the manuscript.

## Abbreviations

AEA: arachidonylethanolamide; Akt/pAkt: Akt kinase/phospho-Akt kinase; CB1: cannabinoid receptor type 1; CB2: cannabinoid receptor type 2; ERK1/2: extracellular receptor-stimulated kinase 1/2; G-6-PD: glucose-6-phosphate dehydrogenase; MAPK: mitogen activated protein kinase; NAE: N-acylethanolamine; p38/phospho-p38: mitogen activated protein kinase of 38 kilodaltons/phosphorylated p38; PEA: palmitoylethanolamine; PKA: protein kinase A; tBHP: tert-butyl hydroperoxide
